# CD248^+^ Cancer-Associated Fibroblasts: A Novel Prognostic and Therapeutic Target for Renal Cell Carcinoma

**DOI:** 10.3389/fonc.2021.773063

**Published:** 2021-12-14

**Authors:** Chao Xu, Keying Zhang, Fa Yang, Xiang Zhou, Shaojie Liu, Yu Li, Shanjin Ma, Xiaolong Zhao, Tong Lu, Shiqi Lu, JiaYu Zhang, Hongji Li, Donghui Han, Weihong Wen, Weijun Qin

**Affiliations:** ^1^ Department of Urology, Xijing Hospital, Fourth Military Medical University, Xi’an, China; ^2^ Department of Clinical Laboratory, Innovation Port Hospital of Xi’an Jiaotong University, Xi’an Jiaotong University, Xi’an, China; ^3^ Department of Urology, Tangdu Hospital, Fourth Military Medical University, Xi’an, China; ^4^ Department of Medical Research, Northwestern Polytechnical University, Xi’an, China

**Keywords:** renal cell carcinoma, CD248, CAFs, TME, prognostic biomarker

## Abstract

**Background:**

The tumor microenvironment (TME) plays an important role in the progression of renal cell carcinoma (RCC). Cancer-associated fibroblasts (CAFs) are considered to constitute a major component of the TME and participate in various tumor-promoting molecular events. We have previously confirmed that CD248 represents a promising biomarker of CAFs, which may provide insight into CAF-based tumor-promoting effects. However, CAF-mediated tumor progression and the potential mechanism of CD248 remain largely unknown in RCC patients.

**Methods:**

Expression profiling and clinical data of RCC patients were obtained from The Cancer Genome Atlas (TCGA) database. An MCP-counter algorithm and Kaplan–Meier survival analysis were performed to explore the prognostic value of CAFs and CD248, respectively. A Pearson correlation coefficient test and Student’s *t*-test were employed to evaluate the relationship between immunosuppressive TME and CD248 or CAFs. Immunohistochemistry and immunofluorescence staining were performed to confirm CD248 expression within CAFs. CD248-specific siRNA was used to investigate the potential function of CD248 in CAF tumor promotion. Differentially expressed genes (DEGs), weighted gene co-expression network analysis (WGCNA), and enrichment analysis were conducted to clarify the function of CD248^+^ CAFs in RCC progression and the associated regulatory mechanism.

**Results:**

CD248 overexpression and CAF infiltration could predict poor RCC prognosis, which may involve the immunosuppressive TME. CD248 may serve as a promising CAFs biomarker and be involved with the tumor-promoting effect of CAFs. Moreover, CD248^+^ CAF infiltration may contribute to RCC progression and an immunosuppressive TME through cell-extracellular matrix (ECM) interactions and metabolism regulation.

**Conclusion:**

CD248^+^ CAFs participate in the regulation of RCC progression and immunosuppressive TME, which may represent a novel prognostic and therapeutic target for RCC.

## Introduction

Renal cell carcinomas (RCCs) are characterized by a high degree of heterogeneity and composed of different subtypes with their own biological properties and therapeutic responses, which lead to different clinical outcomes (1). High infiltration of stromal and immune components and the plasticity of the tumor microenvironment (TME) are the most common features of RCC (2, 3). Like a protective nest, the TME shelters cancer cells by providing vascular nourishment, suppressing immunity, and establishing a matrix barrier (4). Over the past decade, there have been an increased number of innovative therapeutic strategies targeting TME for RCC, among which immune checkpoint blockade (ICIs) and anti-angiogenic tyrosine kinase inhibitors (TKIs) have attracted keen attention (5, 6). However, the acquisition of therapeutic resistance in most patients has limited their further clinical application (7), which is closely correlated with the perfect linkage between tumor parenchymal and TME (8). Consequently, an in-depth understanding of the components of the TME in RCC is conducive to constructing a tumor-specific framework and developing a novel target for RCC therapy.

Cancer-associated fibroblasts (CAFs) have emerged as a potential target to destroy the TME network, given the cross-linkage role of CAFs in tumor initiation and progression (9). Once the tumor is established, multiple CAF-associated pro-tumor factors begin to appear, in which CAFs are both synthesized and remodeled into the extracellular matrix (ECM) to support tumor proliferation and metastasis, and also function as regulators of neo-angiogenesis, immunoregulation, and therapeutic resistance (10). Histopathological analysis also demonstrated that the infiltration of CAFs and CAF-related signals were predictive of a poor prognosis (11, 12). However, due to the non-specificity of conventional biomarkers, such as αSMA, FAP, PDGFRα/β, and FSP1 (S100A4) (13), a deficiency of specific biomarkers to distinguish CAFs from other components in the TME presents a challenge to the identification of potential functions and potential as a target for RCC treatment.

CD248, also known as endosialin or tumor endothelial marker 1 (TEM1), is rarely expressed in normal tissues and is primarily expressed on stromal cells, including activated fibroblasts, as well as the perivascular cells of tumors or inflammatory diseases (14). Recent reports have focused on CD248 as a specific biomarker of activated fibroblasts, including CAFs in tumors and myofibroblasts in organ fibrosis (15–18). Studies have confirmed that CD248 was overexpressed in CAFs of hepatocellular carcinoma (HCC), which promoted the polarization of tumor-associated macrophages (TAMs) to the M2 phenotype, an inhibitory maker in TME, by regulating growth arrest-specific protein 6 (GAS6) (19). In addition, the knockdown of CD248 in mice showed significant suppression mediated by stromal cells in tumor growth, invasion, and metastasis following tumor transplantation (20). Therefore, the potential role of CD248 as a specific biomarker for CAFs makes it an ideal target for the TME of RCCs. However, the characteristics of CD248^+^ CAFs in RCC progression and the associated regulatory mechanisms remain largely unknown.

The aim of the present study was to uncover the role that CD248^+^ CAFs play in the TME of RCCs. Clinical specimens and expression profile assays from the Cancer Genome Atlas (TCGA) and Gene Expression Omnibus (GEO) were analyzed to identify the contribution of CD248 to RCC progression. We found that CD248 expression is highly correlated with CAF infiltration in the TME. Using immunostaining techniques, we confirmed that CD248 was specifically located on CAFs. We also found that CD248 participated in stromal-related signal regulation and the knockdown of CD248 weakened the invasive ability of fibroblasts. Finally, we evaluated the prognostic value of CD248^+^ CAFs in RCC and analyzed their related genes with a weighted gene co-expression network analysis (WGCNA) and enrichment analysis to explore the underlying mechanism of CD248^+^ CAFs in regulating the TME of RCC.

## Materials and Methods

### Data Source and Processing

A total of 895 RCC samples and 128 normal samples were downloaded from the TCGA database (https://portal.gdc.cancer.gov/). Transcriptomic data (Fragments Per Kilobase Million [FPKM]) and the clinical information were integrated using ID numbers. For samples from the same patients, gene expression was averaged using *limma* package of R software. All data were processed by R software (https://www.r-project.org/). In addition, the gene expression profiling dataset (GSE167093) was downloaded from the GEO database (https://www.ncbi.nlm.nih.gov/geo/), including 152 pairs of RCC and para-cancer tissues, and an additional 450 RCC samples. Moreover, the RNA-sequencing dataset for si-CD248 and si-Con (PRJNA608053) was downloaded from the National Center for Biotechnology Information (NCBI) (https://www.ncbi.nlm.nih.gov/).

### CAF Calculation and TME Estimation

MCP-counter algorithm (21) was performed to calculate the CAF scores of patients from the TCGA cohort. Moreover, the CAFs with high CD248 expression were defined as CD248+ CAF and the CAFs with low CD248 expression were defined as CD248- CAF. The median of CD248 expression was employed as the cutoff value. The ESTIMATE algorithm in the estimate package of the R software was employed for TME analysis (22). Three scoring forms, including stromal score, immune score, and ESTIMATE score, corresponded to the proportion of stromal and immune components as well as the sum of both. Therefore, the calculated score could reflect the composition of TME.

### Survival Analysis

A Kaplan–Meier survival analysis was performed to evaluate the prognostic value of the variables, including CD248 expression, stromal, and immune scores; CAF infiltration score; and the CD248^+^ CAF infiltration score. The TCGA-KIRC and KIRP cohorts were further analyzed to validate the effect of CD248^+^ CAFs on the patient survival outcomes.

### Enrichment Analysis

Using gene set enrichment analysis (GSEA) software (version 4.1.0), the Hallmark and C2 Kegg gene set v7.2 were used for the enrichment analysis. Gene sets with NOM *p* < 0.05 and false discovery rate (FDR) *q* < 0.05 were considered to be significant. The “clusterprofiler” R package was used to perform GO and KEGG enrichment analyses. GO terms and KEGG pathways with a *p*-value < 0.05 were considered to be significantly enriched.

### WGCNA of CD248^+^ CAF-Related Differentially Expressed Genes

To obtain the DEGs from the TCGA cohort, we divided the samples into two groups according to the medium level of CD248 expression and CAF score. The differential gene expression between these two groups was performed using R software. Genes with an adjusted *p*-value < 0.05 and |log_2_ FC| > 1 were considered to be significant. The transcription factor (TF) list was retrieved from the Cistrome website (https://cistrome.org), and differentially expressed TFs (DEFs) were identified by matching with the DEGs. WGCNA R package was used for the co-expression analysis, and the most significant modules were selected for an enrichment analysis. Cytospace software (version 3.6.0) was employed to visualize the protein–protein interaction (PPI) networks.

### Cell Culture and Transfection

HFL-1 cells were purchased from the Chinese Academy of Science (Shanghai, China). The cellular identities were confirmed by STR profiling, and all cell lines underwent routine mycoplasma testing. Cells were maintained in DMEM/F12 (Gibco) supplemented with 10% FBS (Gibco) and 1% penicillin-streptomycin (Invitrogen). Transfections were performed by applying OPTI-MEM and Lipofectamine 3000 in accordance with the manufacturer’s instructions. si-CD248 and si-Con were purchased from GenePharma (Shanghai, China) and introduced into the cells at a concentration of 50 nM. The transfected cells were harvested at 36 h post-transfection.

### Western Blotting

The total protein samples were prepared from cell pellets and the concentration was determined using a Bradford assay (ThermoFisher Scientific). Equal amounts of the protein samples were loaded on 10% sodium dodecyl sulfate-polyacrylamide gel (SDS-PAGE) and transferred onto a polyvinylidene fluoride (PVDF) membrane (ThermoFisher Scientific). The membrane was blocked with 5% skim milk, and incubated with the following primary antibodies overnight at 4°C: anti-CD248 (1:1,000, Abcam, #ab48185) and anti-GAPDH (1:2,000, Proteintech, #10494-1-AP). The membranes were next incubated with horseradish peroxidase (HRP)-conjugated secondary antibodies for 1 h at room temperature prior to visualization.

### RT-qPCR

Cells were harvested and the total RNA was isolated with TRIzol. Reverse transcription (RT) was conducted using PrimeScript™ RT Master Mix (TaKaRa, Japan). Next, qPCR was performed using an SYRB Green II kit (#DRR041A; TaKaRa, Japan). The following primers were used: homo-CD248-forward (F): 5’-CTCAACCAACTATCCCCAAGTC-3’ and reverse (R): 5’-GCCTGGGTTCTGATACCTGG-3’; homo-GAPDH-F: 5’-AGGTCGGTGTGAACGGATTTG-3’ and R: 5’-TGTAGACCATGTAGTTGAGGTCA-3’.

### Cell Invasion Assays

The invasive ability of HFL-1 cells was determined using a Transwell assay. HFL-1 cells transfected with si-CD248s or si-Con were seeded into the upper chamber. The DMEM medium supplemented with serum was placed into the lower chamber. The cells on the lower side of the filters were defined as invasive cells.

### Tissue Staining

The paraffin-embedded tissue used for the present assay was collected from the patients undergoing a radical operation at our institute. The subjects provided written informed consent. All protocols were authorized by the Ethics Committee of The First Affiliated Hospital of Air Force Military Medical University. For immunohistochemistry (IHC) staining, after deparaffinization and rehydration, sections were applied for antigen retrieval in a citrate solution. After blocking in goat serum (MXB, China) for 40 min, the sections were incubated with a primary CD248 antibody (Abcam, #ab204914) in a humidified box overnight at 4°C. Next, the sections were incubated with the corresponding HRP-labeled secondary antibodies (Abcam, #ab05718) for 30 min at room temperature prior to visualization with a 3,3’-Diaminobenzidine system (MXB, China). Sections were counterstained with hematoxylin and dehydrated in ascending concentrations of ethanol followed by clearance with xylene. The samples were observed under a Nikon light microscope after being sealed onto slides with neutral balsam. For immunofluorescence (IF) staining, the samples were blocked in goat serum for 1 h at room temperature, followed by an incubation with the following primary antibodies: anti-CD248 (Abcam, #ab204914) and anti-α-SMA (Invitrogen, #MA1-06110). The sections were then incubated with Alexa Fluor 488 (Abcam, #ab150077)- and Alexa Fluor 594 (Abcam, #150117)-conjugated secondary antibodies. The nucleus was counterstained with DAPI before being sealed with glycerin. For Masson’s staining, the sections were stained with corresponding reagents according to the manufacturer’s protocol after deparaffinization and rehydration (Servicebio, China).

### Statistical Analysis

The statistical significance of the mean value of the variables between the two groups was calculated using an unpaired Student’s *t*-test or Pearson’s correlation coefficient test using R software. All data were presented as the mean ± standard error of the mean (SEM) of at least three independent experiments. Differences were considered significant at a threshold of *p* < 0.05.

## Results

### CD248 Upregulation Is Correlated With RCC Deterioration and a Tumor-Promoting TME

**Figure 1:**
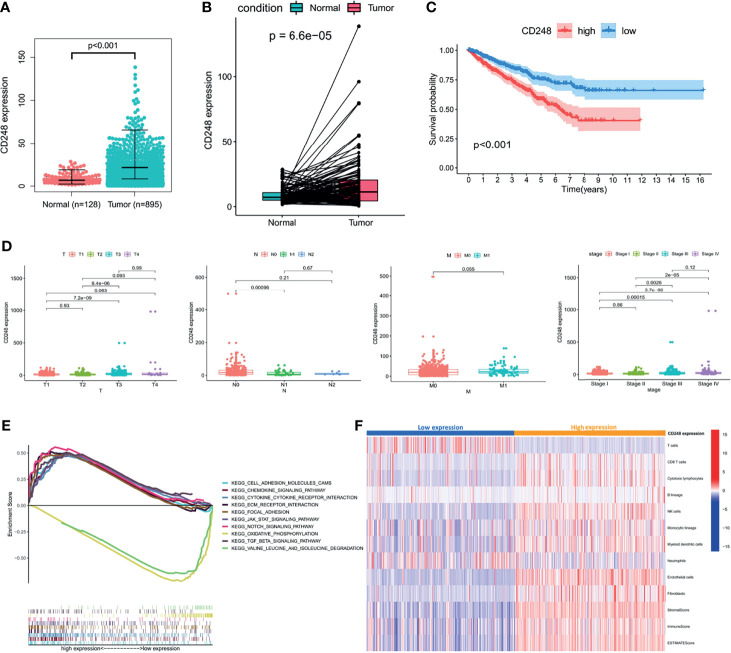
The role of CD248 in RCC progression. **(A, B)** CD248 overexpression in RCC. **(C)** Kaplan–Meier analysis of the OS in high and low CD248-expressing patients with RCC in the TCGA cohort. **(D)** Clinicopathological correlation of CD248 in RCC. **(E)** GSEA of KEGG pathways analysis. **(F)** Heatmap of TME cells was shown after grouping by CD248. *p* < 0.05 was considered statistically significant.

In the TCGA cohort, we compared CD248 expression between normal (*n* = 128) and tumor (*n* = 934) samples from RCC patients and found a significant upregulation of CD248 expression (*p* < 0.001, **Figure 1A**). The normal-tumor paired samples from the same patients yielded a consistent result (*n* = 128, *p* < 0.0001, **Figure 1B**). Likewise, CD248 expression in 152 RCC samples was also significantly higher than that of 152 paired normal samples in the GEO cohort (*p* < 0.001, **Figure S1A**). Moreover, according to the median expression of CD248, the overall survival (OS) rate of RCC patients in the CD248 high-expression group at 5 years was 57.5%, compared with 74.9% for the low-expression group (*p* < 0.001, **Figure 1C**). The clinicopathological correlation analysis indicated that CD248 expression was upregulated as the RCC progressed (*p* < 0.05, **Figure 1D** and **Figure S1B**).

A GSEA of the KEGG pathway was used to analyze the pathways located downstream of CD248 (**Figure 1E**). The results showed that CD248 was positively associated with the activation of pathways related to TME remodeling, including the epithelial–mesenchymal transition (EMT), stromal and ECM signals, immunoregulation, and neo-angiogenesis signals. CH248 was also associated with an inhibition of metabolism-related pathways (*p* < 0.05). An MCP-counter algorithm was performed to determine the relationship between upregulated CD248 and the TME. As a result, the infiltration of tumor epithelial cells, cytotoxic T cells, natural killer (NK) cells, monocytic macrophages, myeloid dendritic cells (MDCS), endothelial cells (ECs), and CAFs was increased in the CD248 high-expression group (**Figure 1F**).

To explore the impact of the TME on RCC prognosis, stromal or immune scores (i.e., a commonly used index to represent the TME) were calculated through the ESTIMATE algorithm. As shown in **Figure S2**, deteriorated tumor progression and a reduced OS rate were detected in the high-score group compared with the low-score group, according to the median scores (*p* < 0.05). Briefly, both CD248 expression and CD248-related TME could predict a poor prognosis.

### CD248 Upregulation Is Correlated With a CAF-Mediated Tumor-Promoting Effect

**Figure 2:**
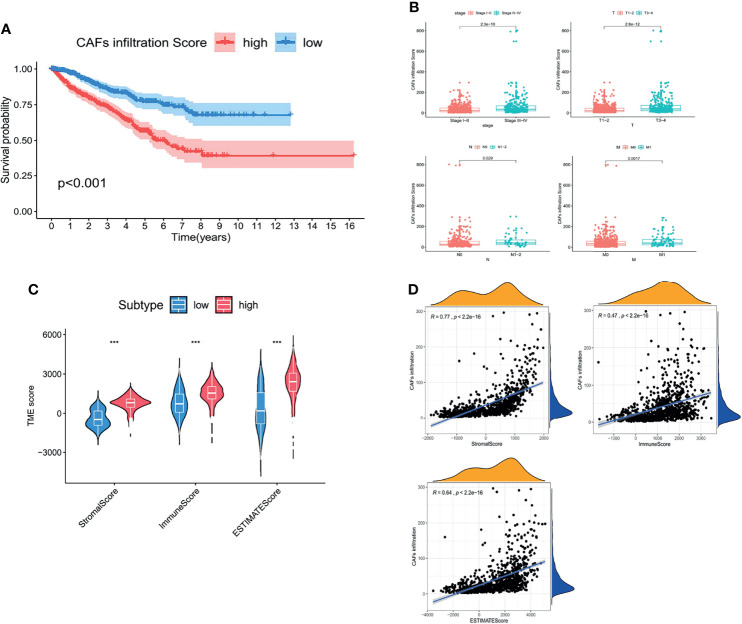
CAFs constituted the major component of the TME and promoted RCC progression. **(A)** Kaplan–Meier analysis of the OS associated with high and low CAF infiltration. **(B)** Clinicopathological correlation of CAF infiltration in RCC. **(C)** Violin plot of the comparison of stromal and immune and ESTIMATE scores based on high and low CAF infiltration groups. **(D)** Correlation analysis between the CAF infiltration score and TME scores. *p* < 0.05 was considered statistically significant.

As the major component of the TME, CAFs participate in various tumor-prompting molecular events. In the present study, the high CAF infiltration group showed a poorer prognosis compared to that of the low infiltration group with the OS rate at 5 years (56.4% and 78.1%, respectively) according to the median CAF infiltration score (*p* < 0.001, **Figure 2A**). Furthermore, RCC patients with a high CAF infiltration have a significantly higher pathological stage and risk of metastasis compared to those in the low infiltration group (*p* < 0.001, **Figure 2B**). Moreover, CAF infiltration was positively associated with the TME score (*p* < 0.05, **Figure 2C**; *p* < 0.001, **Figure 2D**).

**Figure 3:**
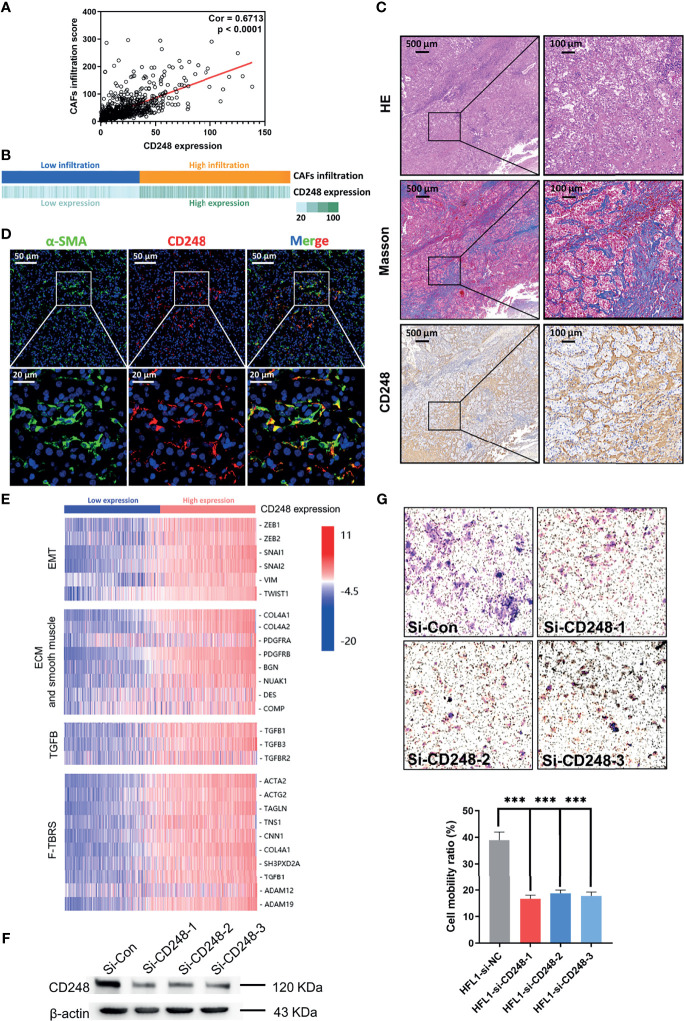
CD248 is a specific biomarker for CAFs. **(A)** Correlation analysis between CD248 expression and CAF infiltration. **(B)** Correlation bar chart between CD248 expression and CAF infiltration. **(C)** H&E, Masson, and IHC staining for CD248 in RCC tumor lesions. Scale bar = 500 μm. **(D)** Dual IF staining showing the colocalization of α-SMA (green) and CD248 (red) in RCC tumor lesions. Scale bar = 50 μm. **(E)** Hierarchical clustering in the TCGA cohort. **(F)** Western blot showing the effective knockdown of CD248 in the HFL-1 cell line. **(G)** Images of the Transwell assay results following the knockdown of CD248 in HFL-1 cell lines, and representational statistical analysis of the Transwell assay. *n* = 3. Data are shown as the mean ± SEM. Representative images are shown. *p* < 0.05 was considered statistically significant. ***p < 0.001.

Considering that the prognostic value of both CD248 and CAFs was related to the TME, together with our previous findings that CD248 contributes to tumor-promoting regulation of CAFs in HCC (19), we proposed to explore the relationship between CD248 and CAFs in RCC progression. **Figures 3A, B** show that CD248 expression was significantly associated with CAF infiltration (Cor = 0.67, *p* < 0.0001). Using RCC tissue staining, we observed that the expression pattern of CD248 and collagen (mainly CAF synthesis) was similar in the tumor interstitium (**Figure 3C**). Furthermore, CD248 was colocalized with α-SMA (a CAF biomarker) in the RCC sections (**Figure 3D**). Hierarchical clustering results showed that upregulated CD248 was positively correlated with mesenchymal activation, including EMT, ECM-related signals, TGFβ signals, and pan-fibroblast TGFβ response (F-TBRS) in TCGA RCC patients (**Figure 3E**). HFL-1 cells (a fibroblasts cell line) with CD248 knocked down showed a decreased-invasion ability in a Transwell assay (*p* < 0.0001, **Figure 3F** and **Figure S3A**; *p* < 0.001, **Figure 3G**), which indicated that CD248 might contribute to a CAF-mediated tumor-promoting effect.

### Regulatory Mechanism of CD248 in CAF-Mediated Tumor-Promoting Molecular Events

**Figure 4:**
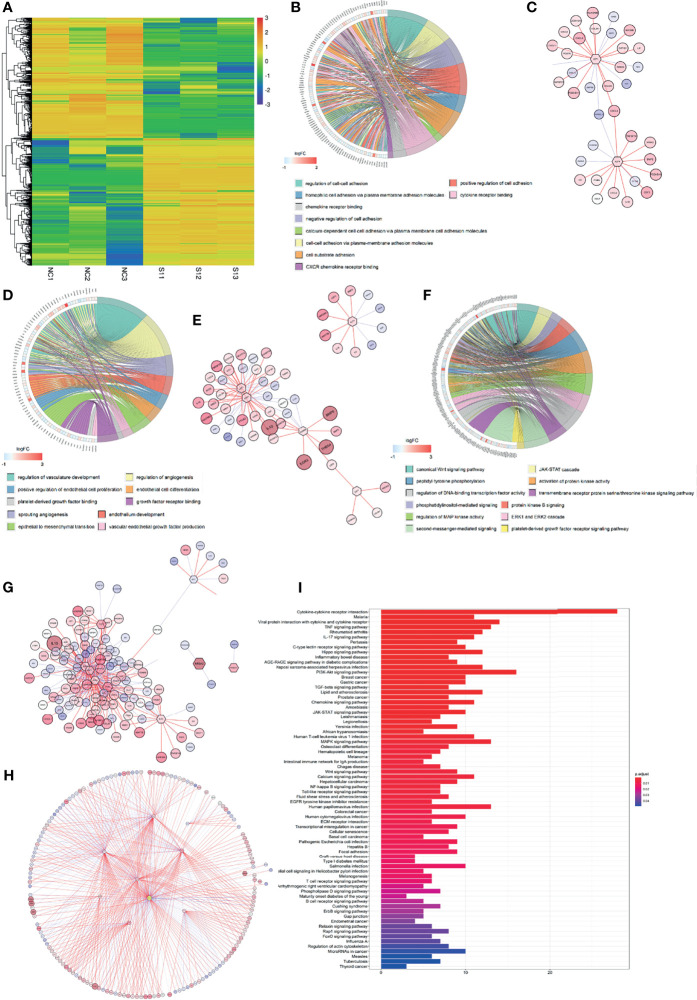
CD248 promotes the role of CAFs in the TME. **(A)** Hierarchical clustering in the RNA-seq from the CD248 knockdown HFL-1 cell line. **(B, D, F)** Chord charts for the GO enrichment analysis. The left semicircle represents the genes and the right semicircle represents the functions of these gene sets involved in regulation. **(C, E, G)** PPI networks for each of the gene sets. **(H)** The total PPI network. **(I)** KEGG pathways enrichment analysis. *p* < 0.05 was considered to be statistically significant.

The RNA-Seq profile of HFL-1 cells (i.e., control group and CD248 knockdown group) was analyzed using R software. From this process, 2,104 DEGs were obtained, from which 1,084 genes were upregulated, and 1,019 genes were downregulated in the CD248 knockdown group compared with the control group (FDR < 0.05, |log_2_ FC| > 1, **Figure 4A**). Subsequently, 1,519 DEGs were correlated with CD248 expression and 32 DEFs were selected (Cor = 0.5, *p* < 0.05). The GO enrichment analysis showed that CD248-related genes were primarily enriched in three types of functions, including cell–cell and cell–cytokine interactions, tumor angiogenesis, and cellular proliferation. Chord plots and corresponding PPI networks were constructed for visualization (FDR < 0.05, **Figures 4B−G**). Moreover, an integrated PPI of those three above sub-networks was constructed to explore the regulatory mechanism between different GO terms (**Figure 4H**). As a result, several hub DEFs with maximum intramodular connectivity were identified (e.g., SOX4, EGR1, LEF1, FOS, MITF, KLF4, TCF7, and KDM6B), which might involve CD248-mediated tumor-promoting regulation in CAFs (**Figure 4H**). A KEGG analysis of the integrated PPI was performed, and immunoregulation, tumor progression, and neo-angiogenesis pathways were significantly enriched (FDR < 0.05, **Figure 4I**), among which the cytokine–cytokine receptor interaction, PI3K-Akt signaling pathway, and MAPK signaling pathway exhibited the maximum intramodular enrichment.

### CD248^+^ CAFs Are Associated With an Immunosuppressive TME in RCC Progression

**Figure 5:**
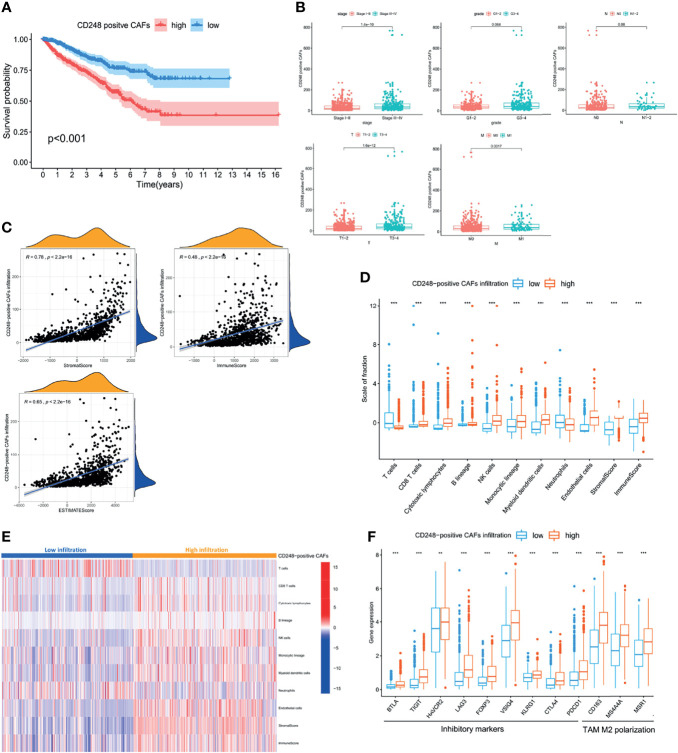
CD248^+^ CAF infiltration was suggestive of a poor prognosis in RCC patients and immunosuppression. **(A)** Kaplan–Meier analysis of the OS associated with high and low CD248^+^ CAF infiltration. **(B)** Clinicopathological correlation of CD248^+^ CAF infiltration in RCC. **(C)** Correlation analysis between the CAF infiltration score and TME scores. **(D)** Box plot of the comparison of cells in the TME based on high and low CD248^+^ CAF infiltration groups. **(E)** Heatmap of TME cells shown after grouping by CD248^+^ CAFs. **(F)** Box plot of the comparison of inhibitory immune molecules based on high and low CD248^+^ CAF infiltration groups. *p* < 0.05 was considered statistically significant. **p < 0.01; ***p < 0.001.

To further investigate the function of CD248 in CAF-mediated tumor promotion, CAFs exhibiting upregulated CD248 were defined as CD248^+^ CAFs according to the median expression level. As shown in **Figure 5A**, a high infiltration of CD248^+^ CAFs in RCC patients was related to a poorer OS. In particular, the OS rate at 5 years was 58.3% in the high infiltration group, whereas this index was 77.5% in the low infiltration group (*p* < 0.001). Additionally, increased CD248^+^ CAF infiltration could indicate worse tumor stage, local invasion, and metastasis of RCC (*p* < 0.01, **Figure 5B**). The similar prognostic value of CD248^+^ CAFs in the two major subtypes of RCC (i.e., clear-cell RCC and papillary RCC) were further validated in TCGA-KIRC and KIRP cohorts (*p* < 0.01, **Figures S4A, B**).

Since both CD248 and CAFs were associated with immunoregulation, the CD248^+^ CAF-mediated RCC tumor-promoting effect may be involved. Moreover, CD248^+^ CAF infiltration was positively correlated with the TME scores, particularly the immune score (*p* < 0.0001, **Figure 5C**). The results of the MCP-counter algorithm revealed that increased CD248^+^ CAF infiltration was accompanied by a higher infiltration of CD8^+^ T cells, NK cells, monocytic macrophages, and MDCS (*p* < 0.001, **Figures 5D, E**). However, the expression level of several T-cell depletion biomarkers (i.e., PDCD1, CTLA4, LAG3, FOXP3, and HAVCR2) and TAM type 2 polarization biomarkers (i.e., CD163, MS4A4A, and MSR1) were significantly increased in the high CD248^+^ CAF group (*p* < 0.001, **Figure 5F**). This indicates that CD248^+^ CAFs might be associated with an immunosuppressive TME in RCC progression.

### Regulatory Mechanism of CD248^+^ CAFs in RCC Immunosuppression

**Figure 6:**
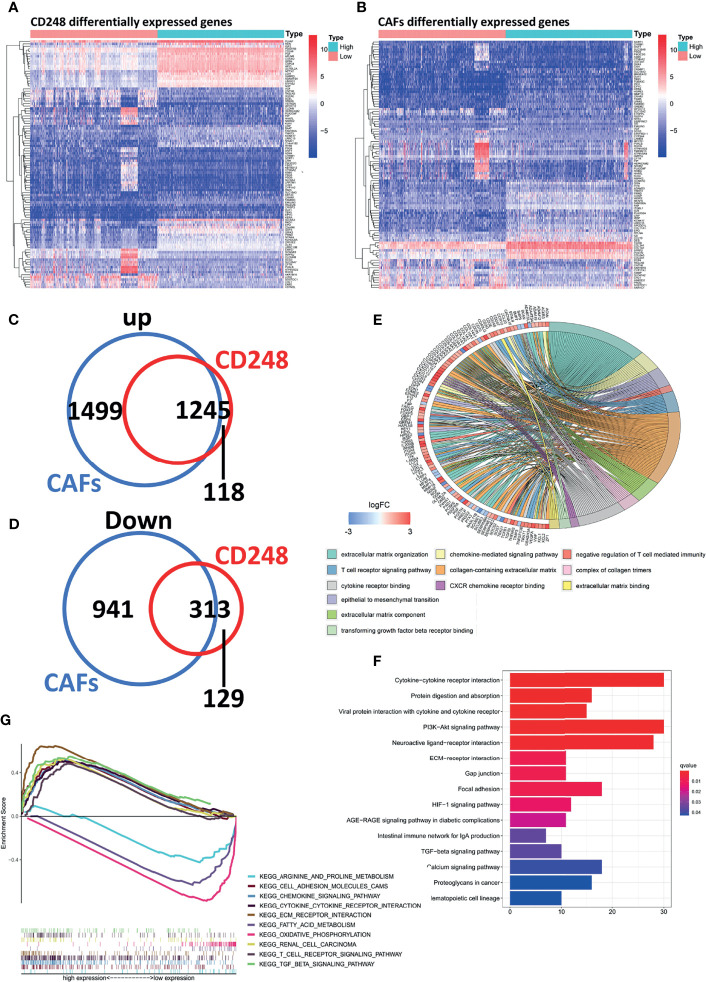
Recognition of regulatory DEGs for CD248^+^ CAFs and enrichment analysis. **(A)** Heatmap showing DEGs based on median CD248 expression. **(B)** Heatmap showing DEGs based on the median CAF infiltration. **(C, D)** The intersection of DEGs associated between CD248 and CAFs. **(E)** GO function enrichment analysis. **(F)** KEGG pathway analysis. **(G)** GSEA of KEGG pathway analysis. *p* < 0.05 was considered statistically significant.

A total of 3,186 and 2,617 DEGs that correlated with both CD248 and CAFs were recognized in the TCGA cohort, respectively (FDR < 0.05, |log_2_ FC| > 1, **Figures 6A, B**). CD248^+^ CAF-correlated DEGs were identified, among which 1,245 were upregulated and 313 DEGs were downregulated (**Figures 6C, D**). Through a GO and KEGG enrichment analysis, those CD248^+^ CAF-correlated DEGs were mainly enriched in the activation of immunoregulation pathways, cell–cell and cell–ECM interactions, and inhabitation of metabolism-related pathways (i.e., oxidative phosphorylation and amino acid metabolism) (*p* < 0.05, **Figures 6E–G**).

**Figure 7:**
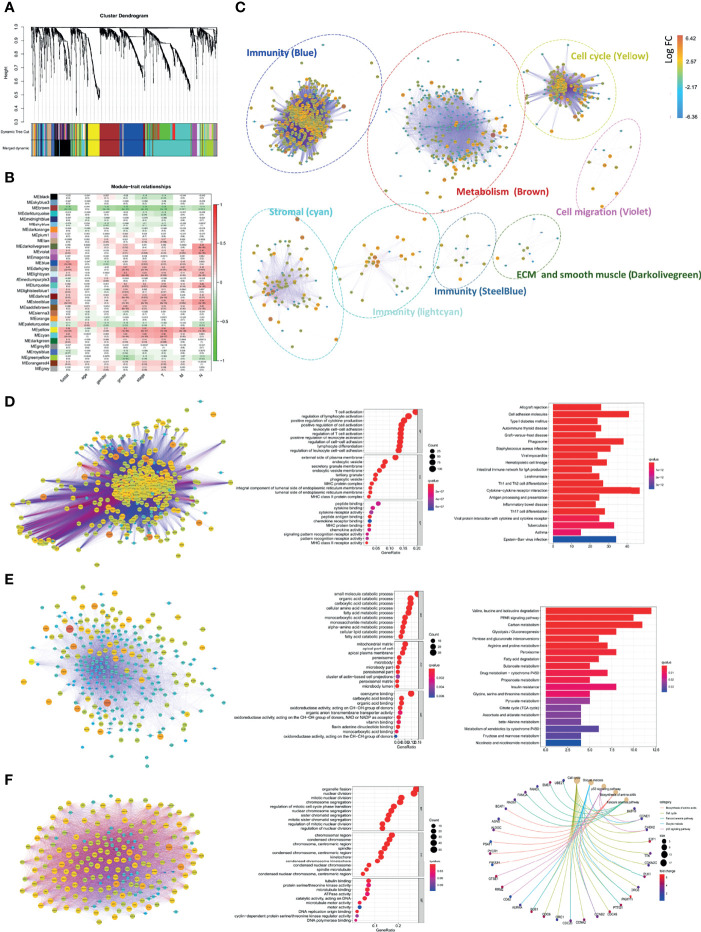
Comprehensive annotation for the functions of CD248^+^ CAFs regulatory DEGs. **(A)** Clustering dendrograms of genes and clinicopathological variables, with dissimilarity based on topological overlap, together with assigned module colors. **(B)** Module–trait associations. The rows correspond to module gene sets, and columns correspond to a trait. Cells contain the corresponding correlation and *p*-value. The table is color-coded by correlation according to the color legend. **(C)** PPI network for the 8 selected gene modules. The function of each module is marked. Labels are colored according to the color legend. **(D)** PPI network and bubble diagram of the GO analysis and bar chart of KEGG pathway analysis for the “Blue” module. **(E)** PPI network and bubble diagram of the GO analysis and bar chart of KEGG pathways analysis for the “Brown” module. **(F)** PPI network and bubble diagram of the GO analysis and Circos chart of the KEGG pathway analysis for the “Yellow” module. *p* < 0.05 was considered statistically significant.

To accurately identify hubs of gene clusters of immunoregulation and tumor promotion, 1,558 CD248^+^ CAF-correlated DEGs were analyzed through the WGCNA method (**Figure 7A** and **Figures S5A, B**). These 1,558 DEGs were then categorized into 32 modules (i.e., gene clusters), and we selected eight modules that were significantly associated with clinicopathological variables (Cor > 0.3, *p* < 0.001, **Figure 7B**). GO terms and KEGG pathway enrichment analysis were employed for the functional annotation of the eight modules (**Figure 7C**). Apart from the classical functions of CAFs in ECM remodeling, cell–ECM interactions, and cellular migration (**Figures S6A–C**), CD248^+^ CAF-correlated DEGs were also enriched in the terms of immune cell activation and cell–cytokine interactions (**Figure 7D** and **Figures S6D–E**). In addition, the metabolism process (i.e., fatty acid, glycolysis, and amino acid metabolism) and cell cycle (i.e., promoting mitosis and relieving proliferation inhibitory signals) terms were significantly enriched (**Figures 7E, F**). For visualization, corresponding PPI networks of each module were simultaneously constructed.

## Discussion

The incidence of RCC has increased annually, accounting for approximately 90%−95% of neoplasms in the kidneys (23). Among patients with RCC, about 40% of patients miss surgery due to the occult progression of the tumor (24). In addition, despite undergoing timely surgical treatment, approximately 28% of patients experience relapse or develop metastasis with a low 5-year survival (25). Fortunately, therapeutic strategies for RCC have rapidly evolved throughout the last decade, among which ICIs or TKIs alone or in combination have been regarded as the standard treatment in advanced RCC (26); however, a moderate tumor mutation burden (TMB) and an inverse correlation between infiltrating CD8^+^ T cells and prognosis of patients have limited the further utility of ICIs (27, 28). Moreover, a low complete response rate and the activation of angiogenic escape pathways decreased the therapeutic expectation of TKIs (29). Apparently, the complexity of TME brings new anti-tumor targets, but also hides the potential therapeutic resistance mechanism. Therefore, the identification of stable and effective targets from a dynamic alteration of TME will be of central significance for RCC treatment.

As the dominant member of the TME, CAFs participate in all stages of tumor progression, including tumorigenesis and metastasis (30), in which CAFs support the TME *via* cell–cell interactions and the secretion of regulators to promote neo-angiogenesis, accumulate inhibitory immune cells, remodel the ECM, and promote EMT, including vascular endothelial growth factor A (VEGFA), TGFβ, CXCL12, CCL2, IL6, and MMPs, among others (31). Therefore, targeting CAFs to transform the TME from being tumor-promoting to tumor-suppressive represents a recent research hotspot and effective strategy. However, the limitations of specific biomarkers for the identification of CAFs prevent us from further understanding the biology and functions of CAFs in tumorigenesis. CD248 is a single transmembrane glycoprotein that has been reported as a specific biomarker of activated fibroblasts, which is rarely expressed in normal tissues and is upregulated in the stroma of tumors and chronic inflammatory diseases, enabling it to function as a specific biomarker for CAFs (18, 32). In the present study, we first recognized that CD248 overexpression was associated with adverse prognostic outcomes and disease progression in RCC. The GSEA of KEGG pathway analysis revealed that CD248 participated in TME-related signatures, including stroma-related, cell–cell, and cell–ECM interactions, immunoregulation, and EMT signatures. In addition, CD248 overexpression was accompanied by increased infiltration of CD8^+^ T cells, NK cells, monocytic macrophages, and MDCS, which confirmed that CD248 has a remarkable capacity to recruit immune cells. Since high stromal and immune cell infiltration is associated with a poor prognosis in RCC patients, CD248 may influence RCC progression by regulating the TME.

We next identified that the high infiltration of CAFs was inversely correlated with the clinical outcomes of RCC patients and positively associated with the complexity of the TME, suggesting that CAFs play a dominant role in the TME. Since CD248 expression in CAFs has previously been reported (18, 19), when coupled with CD248-related signatures significantly enriched in the stroma and ECM pathways, we suspected that the upregulation of CD248 might correlate with the CAF-mediated tumor-promoting effect. Indeed, a high correlation between CD248 expression and CAF infiltration was detected in the present study. Additionally, CD248 was significantly accumulated in the ECM enriched region, and was colocated with αSMA, which is a reliable biomarker for fibroblasts (33). CD248 overexpression indicated the activation of stromal-related signatures (e.g., EMT), cell–ECM interactions, TGFβ, and F-TBRS-related signals, which were highly involved in CAF functions. Furthermore, the knockdown of CD248 weakened the invasive ability of fibroblasts. Through the analysis of the differentiation between si-CD248 and si-Con-treated HFL1 cells, we found that fibroblasts could regulate cytokine release and related receptor expression through CD248, and participate in matrix adhesion, angiogenesis, and other functions.

To further investigate the function of CD248 in CAF-mediated tumor-promoting regulation, CAFs with upregulated CD248 were defined as CD248^+^ CAFs. We initially determined that the high infiltration of CD248^+^ CAFs in the TME decreased the OS rate of RCC patients and were indicative of RCC deterioration. Moreover, the increase of CD248^+^ CAFs in the tumor reflected the complexity of TME components, in which there was an increase in the stromal and immune ingredients and was accompanied by the proliferation of epithelial cells. In addition, CD248^+^ CAFs could recruit CD8^+^ and cytotoxic T cells, as well as promote the infiltration of monocytic macrophages, NK cells, and MDCS. However, immunosuppression is one of the most important characteristics of TME in RCC (34), and increased CD248^+^ CAF infiltration was associated with a suppressive immune microenvironment, in which exhausted T cells and M2-type TAMs were expansively infiltrated. Thus, participation in the suppressive immune microenvironment was one of the reasons that CD248^+^ CAFs lead to a poor prognosis of RCC patients. Furthermore, to comprehensively explore the mechanisms of CD248^+^ CAFs in RCC progression, we identified a total of 1,558 DEGs related to CD248^+^ CAFs. Through the GO and KEGG enrichment analysis, we found that CD248^+^ CAF-correlated DEGs were primarily enriched in the functions and pathways associated with cell–cell and cell–ECM interactions, immunoregulation, and metabolism. To accurately identify the hub gene clusters of immunoregulation and tumor promotion, we next categorized these related DEGs into eight functional modules based on the WGCNA analysis, and selected the most significant modules associated with tumor progression. As a result, CD248^+^ CAFs might influence immunity, stromal-related signatures, cell adhesion, cell cycle, and metabolism to promote tumor deterioration. In particular, CD248^+^ CAFs both contribute to the upregulation of exhausted immune biomarkers and also regulate cytokine and chemokine secretion and expression of related receptors, which resulted in an immunosuppressive TME. Additionally, forming and remodeling the ECM to block normal immune cell infiltration and facilitating the invasion of epithelial cells might be another mechanism by which CD248^+^ CAFs promote tumor progression (35). Nutrient limitation has been shown to be a critical feature of TME (36), and the depression of certain amino acids (e.g., arginine or accumulation of fatty acids) can lead to immunosuppressive effects (37). In the present study, we found that high CD248^+^ CAF infiltration generally leads to metabolic inhibition. Finally, sustaining proliferative signatures and evading growth suppressors to facilitate tumor disordered amplification is another pathway that CD248^+^ CAFs use to facilitate tumor progression.

In summary, CD248^+^ CAFs participate in the regulation of RCC progression and an immunosuppressive TME, which may represent a novel prognostic and therapeutic target for RCC. The antibody (19), antibody–drug conjugates (ADCs) (38), vaccine (39), and even CAR-T cells targeting CD248^+^ CAFs might become promising strategies for the destruction of the cancer nest and overcoming the immunosuppressive TME.

## Data Availability Statement

The original contributions presented in the study are included in the article/**Supplementary Material**. Further inquiries can be directed to the corresponding authors.

## Ethics Statement

The studies involving human participants were reviewed and approved by the Ethics Committee of The First Affiliated Hospital of Air Force Military Medical University. The patients/participants provided their written informed consent to participate in this study.

## Author Contributions

CX, KZ, and FY: data acquisition, data analysis, and writing original draft. CX, KZ, FY, XZ, SJL, XLZ, and HL: methodology, data interpretation, writing review, and editing. WQ, WW, and DH: conceptualization, design, and project administration. All authors contributed to the article and approved the submitted version.

## Funding

This work was partly supported by grants from the National Natural Science Foundation of China (No. 81772734) and the Innovation Capability Support Program of Shaanxi (Program No. 2020PT-021).

## Conflict of Interest

The authors declare that the research was conducted in the absence of any commercial or financial relationships that could be construed as a potential conflict of interest.

## Publisher’s Note

All claims expressed in this article are solely those of the authors and do not necessarily represent those of their affiliated organizations, or those of the publisher, the editors and the reviewers. Any product that may be evaluated in this article, or claim that may be made by its manufacturer, is not guaranteed or endorsed by the publisher.
