# Supplementary material for: CD248+ Cancer-Associated Fibroblasts: A Novel Prognostic and Therapeutic Target for Renal Cell Carcinoma
Source: Front Oncol. 2021 Dec 14;11:773063. doi: 10.3389/fonc.2021.773063 (PMC8712640; doi:10.3389/fonc.2021.773063)
Supplement: Supplementary file 1 [file DataSheet_1.pdf]

SUPPLEMENTARY FIGURES

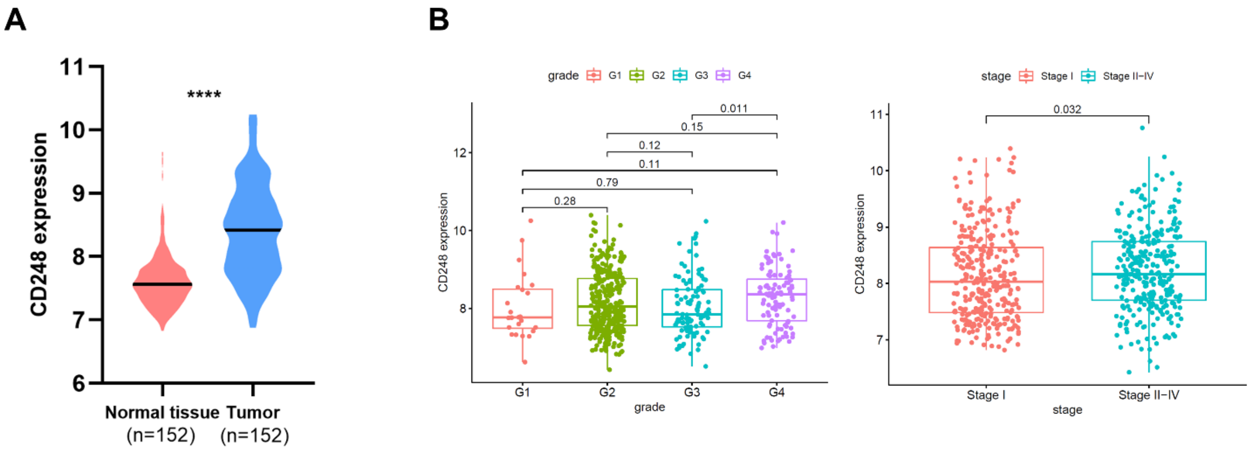

FIGURE S1 | (A) The expression levels of CD248 from GEO cohort (n = 304). **(B)** Clinicopathological correlation of CD248 expression in GEO cohort (n= 602). $P < 0.05$ was considered statistically significant. $^{*}P < 0.05$; $^{**}P < 0.01$; $^{***}P < 0.001$; $^{****}P < 0.0001$.

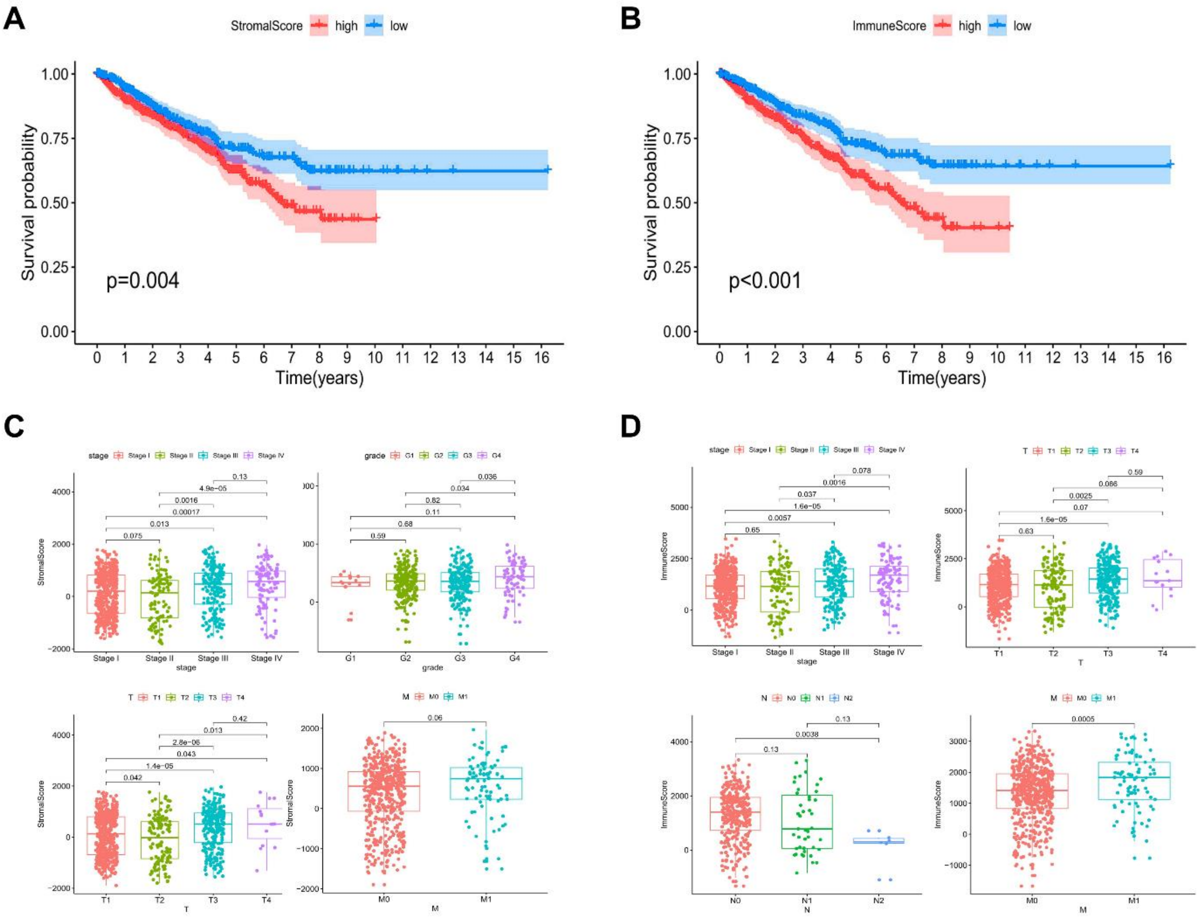

FIGURE S2 | (A) Kaplan-Meier analysis of the OS rate in high and low stromal scores. **(B)** Kaplan-Meier analysis of the OS rate in high and low immune scores. **(C)** Clinicopathological correlation of the stromal score. **(D)** Clinicopathological correlation of the immune score. P < 0.05 was considered statistically significant.

A

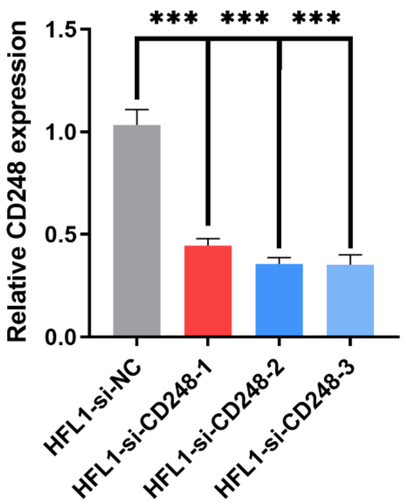

FIGURE S3 | (A) RT-qPCR analysis showing the level of CD248 mRNA expression. Data are shown as the mean ± SEM. $P < 0.05$ was considered statistically significant. * $P < 0.05$; ** $P < 0.01$; *** $P < 0.001$; and **** $P < 0.0001$.

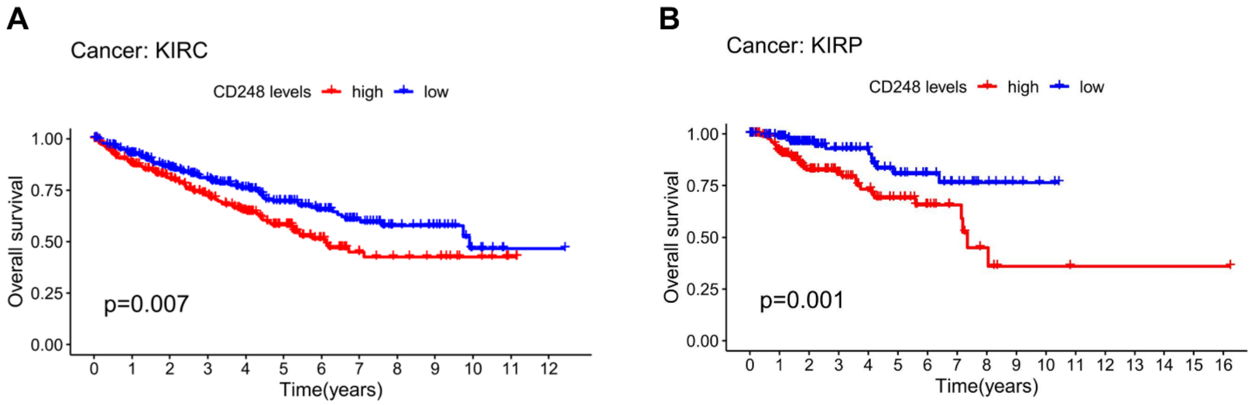

FIGURE S4 | (A) Kaplan-Meier analysis of the OS rate based on $CD248^{+}$ CAF infiltration in the KIRC TCGA cohort. **(B)** Kaplan-Meier analysis of the OS rate based on $CD248^{+}$ CAF infiltration in the KIRP TCGA cohort. $P < 0.05$ was considered statistically significant.

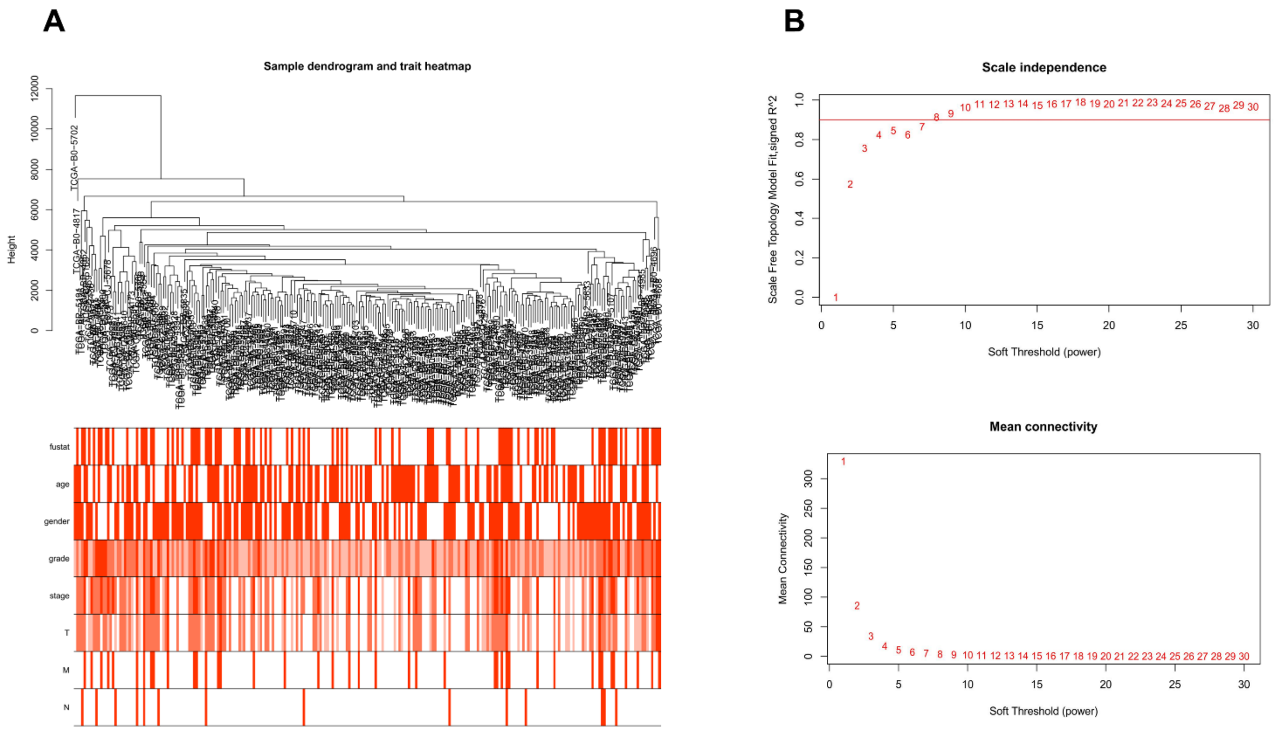

FIGURE S5 | (A) Sample clustering based on gene expression patterns and clinicopathological variables to detect outliers. All of the samples were located in clusters and all samples passed the cuts. **(B)** Analysis of network topology for various soft thresholding powers.

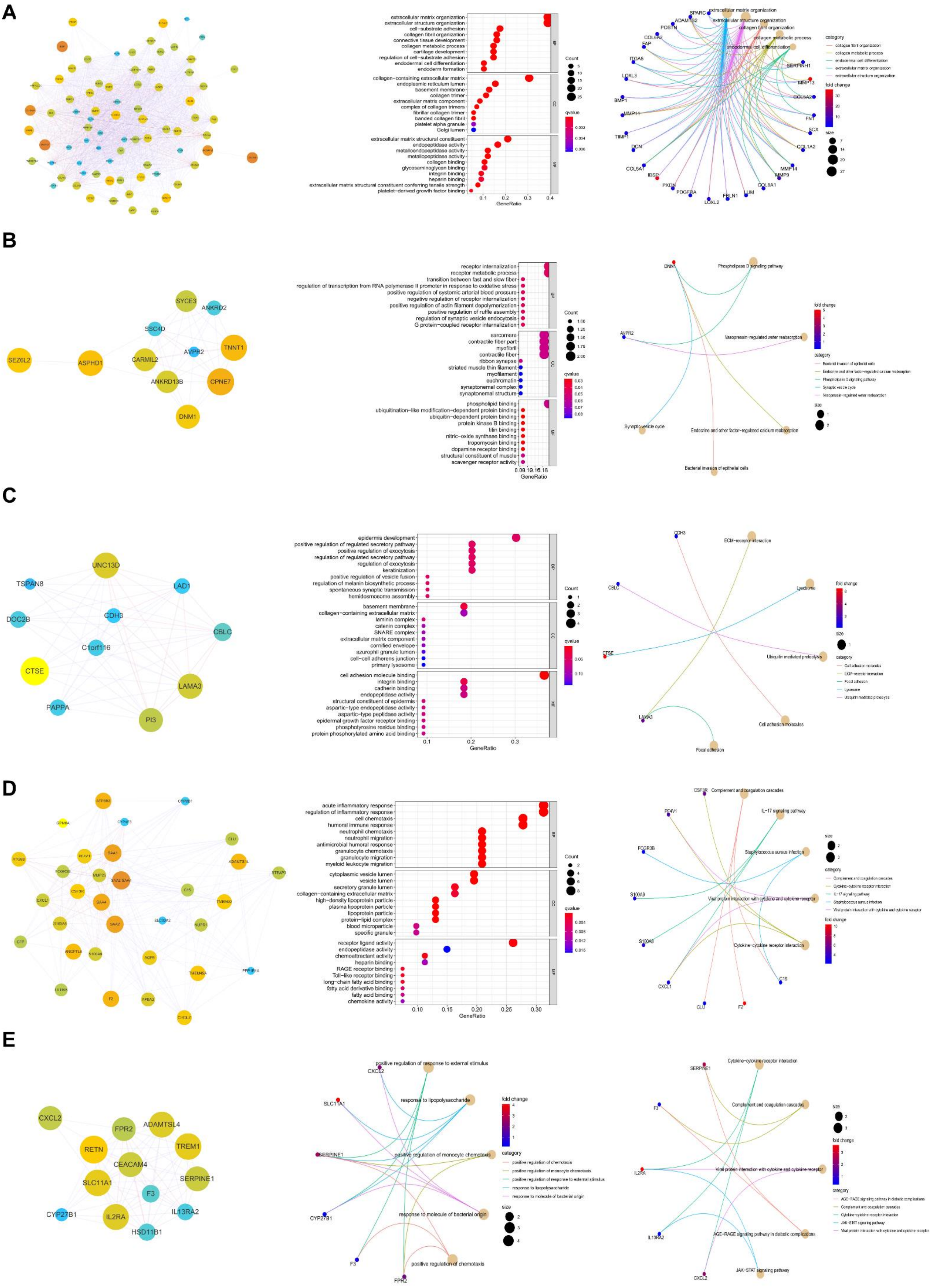
A
extracellular matrix organization
extracellular structure organization
cell−substrate adhesion
collagen fibril organization
connective tissue development
collagen metabolic process
cartilage development
regulation of cell−substrate adhesion
endodermal cell differentiation
endoderm formation
collagen−containing extracellular matrix
endoplasmic reticulum lumen
basement membrane
collagen trimer
extracellular matrix component
complex of collagen trimers
fibrillar collagen trimer
banded collagen fibril
platelet alpha granule
Golgi lumen
extracellular matrix structural constituent
endopeptidase activity
metalloendopeptidase activity
metallopeptidase activity
collagen binding
glycosaminoglycan binding
integrin binding
heparin binding
extracellular matrix structural constituent conferring tensile strength
platelet−derived growth factor binding
GeneRatio
Count
qvalue
SPARC
ADAMTS2
POSTN
COL5A2
FAP
ITGA5
LOXL3
BMP1
MMP11
TIMP1
DCN
COL5A1
IBSP
PXDN
PDGFRA
LOXL2
FBLN1
LUM
COL6A1
MMP9
MMP14
COL1A2
SCX
FN1
COL6A2
MMP13
SERPINH1
category
fold change
size
B
SEZ6L2
ASPHD1
SYCE3
ANKRD2
SSC4D
CARMIL2
AVPR2
TNNT1
ANKRD13B
CPNE7
DNM1
receptor internalization
receptor metabolic process
transition between fast and slow fiber
regulation of transcription from RNA polymerase II promoter in response to oxidative stress
positive regulation of systemic arterial blood pressure
negative regulation of receptor internalization
positive regulation of actin filament depolymerization
positive regulation of ruffle assembly
regulation of synaptic vesicle endocytosis
G protein−coupled receptor internalization
sarcomere
contractile fiber part
myofibril
contractile fiber
ribbon synapse
striated muscle thin filament
myofilament
euchromatin
synaptonemal complex
synaptonemal structure
phospholipid binding
ubiquitination−like modification−dependent protein binding
ubiquitin−dependent protein binding
protein kinase B binding
titin binding
nitric−oxide synthase binding
tropomyosin binding
dopamine receptor binding
structural constituent of muscle
scavenger receptor activity
GeneRatio
Phospholipase D signaling pathway
Vasopressin−regulated water reabsorption
Synaptic vesicle cycle
Endocrine and other factor−regulated calcium reabsorption
Bacterial invasion of epithelial cells
C
UNC13D
TSPAN8
LAD1
DOC2B
CDH3
CBLC
C1orf116
CTSE
LAMA3
PAPPA
PI3
epidermis development
positive regulation of regulated secretory pathway
positive regulation of exocytosis
regulation of regulated secretory pathway
regulation of exocytosis
keratinization
positive regulation of vesicle fusion
regulation of melanin biosynthetic process
spontaneous synaptic transmission
hemidesmosome assembly
basement membrane
collagen−containing extracellular matrix
laminin complex
catenin complex
SNARE complex
extracellular matrix component
cornified envelope
azurophil granule lumen
cell−cell adherens junction
primary lysosome
cell adhesion molecule binding
integrin binding
cadherin binding
endopeptidase activity
structural constituent of epidermis
aspartic−type endopeptidase activity
aspartic−type peptidase activity
epidermal growth factor receptor binding
phosphotyrosine residue binding
protein phosphorylated amino acid binding
GeneRatio
ECM−receptor interaction
Lysosome
Ubiquitin mediated proteolysis
Cell adhesion molecules
Focal adhesion
D
acute inflammatory response
regulation of inflammatory response
cell chemotaxis
humoral immune response
neutrophil chemotaxis
neutrophil migration
antimicrobial humoral response
granulocyte chemotaxis
granulocyte migration
myeloid leukocyte migration
cytoplasmic vesicle lumen
vesicle lumen
secretory granule lumen
collagen−containing extracellular matrix
high−density lipoprotein particle
plasma lipoprotein particle
lipoprotein particle
protein−lipid complex
blood microparticle
specific granule
receptor ligand activity
endopeptidase activity
chemoattractant activity
heparin binding
RAGE receptor binding
Toll−like receptor binding
long−chain fatty acid binding
fatty acid derivative binding
fatty acid binding
chemokine activity
GeneRatio
CSF3R
Complement and coagulation cascades
PF4V1
IL−17 signaling pathway
FCGR3B
Staphylococcus aureus infection
S100A9
Viral protein interaction with cytokine and cytokine receptor
S100A8
Cytokine−cytokine receptor interaction
CXCL1
C1S
CLU
F2
E
CXCL2
FPR2
ADAMTSL4
RETN
TREM1
CEACAM4
SLC11A1
SERPINE1
F3
CYP27B1
IL2RA
IL13RA2
HSD11B1
positive regulation of response to external stimulus
response to lipopolysaccharide
positive regulation of monocyte chemotaxis
response to molecule of bacterial origin
positive regulation of chemotaxis
Cytokine−cytokine receptor interaction
Complement and coagulation cascades
Viral protein interaction with cytokine and cytokine receptor
AGE−RAGE signaling pathway in diabetic complications
JAK−STAT signaling pathway

FIGURE S6 | (A) PPI network and bubble diagram of the GO analysis and circos chart of the KEGG pathway analysis for the "Cyan" module. **(B)** PPI network and bubble diagram of the GO analysis and circos chart of the KEGG pathway analysis for the "Violet" module. **(C)** PPI network and bubble diagram of the GO analysis and circos chart of KEGG pathway analysis for the "Darkolivegreen" module. **(D)** PPI network and bubble diagram of the GO analysis and circos chart of KEGG pathway analysis for the "lightcyan" module. **(E)** PPI network and circos chart of the GO and KEGG pathway analysis for the "SteelBlue" module.
